# Supplementary material for: A UMLS-based spell checker for natural language processing in vaccine safety
Source: BMC Med Inform Decis Mak. 2007 Feb 12;7:3. doi: 10.1186/1472-6947-7-3 (PMC1805499; doi:10.1186/1472-6947-7-3)
Supplement: Additional file 13 — RAP application directory. Sets up directory for RAP (RDF application for PHP) source code [file 1472-6947-7-3-S13.gz › rap/doc/tutorial/netapi.html]

RDF Net API


### RDF API for PHP V0.9.1

# RAP NetAPI - An RDF Server for PHP

This document is part of the RAP - Rdf API for PHP documentation.

Phil Dawes <pdawes@users.sf.net>  
Chris Bizer <chris@bizer.de>   
May 2004

  

## About

The RAP NetAPI is a server for publishing RDF models on the web. Models have
URLs and they can be accessed by query using HTTP GET.

The RAP NetAPI provides an subset implementation of the W3C member submission
RDF
NetAPI. It allows running an RDF server, with similar functionality as the
Joseki RDF server, on web
servers that provide PHP support. It is a simple alternative to Joseki that
can be used in on cheap public webspace.

The RAP NetAPI implementation supports:

- Serving multiple database or file backed RDF models
- RDQL queries using HTTP GET
- FETCH queries using HTTP GET
- SPO queries using HTTP GET
- ADD operation using HTTP POST
- REMOVE operation using HTTP POST
- bNode closure for RDQL, FETCH and SPO
- query result content negotiation (RDF/XML, N3 or N-Triples)

## Installing the NetAPI

1. The first thing you need to do is get mod-rewrite to redirect model queries
   to the netapi.php handler script. To do this you need to make sure that the
   rewrite module is enabled in the Apache configuration file "httpd.conf":  
     

   `LoadModule rewrite_module modules/mod_rewrite.so`

     
   And to make sure that output buffering is turned on in the PHP configuration
   file "php.ini":  
     

   `output_buffering = On`
2. You need to edit the .htaccess file in the netapi directory. Change the
   'RewriteBase' bit to reflect the URI base of the netapi directory. E.g.  
     

   `RewriteEngine on  
   RewriteBase /path/to/rdfapi-php/netapi/ #<---- Edit this   
   RewriteRule ^[^\.]+$ netapi.php`
3. If you want to serve models that are stored in a RAP database, then you
   must edit the config.inc file in the netapi directory to reflect your database
   connection parameters. More Information about creating a RAP database and
   connecting to it is found in the Tutorial:
   Database Persistence.  
     

   `$DB_DRIVER = 'MySQL';   
   $DB_HOST = 'localhost';   
   $DB_DB = 'testrapdb';   
   $DB_USER = 'myuser';   
   $DB_PASS = 'mypass';`
4. Finally, you need to edit the $modelmap array in the netapi/config.inc file.
   This array maps model names to model URIs, and is used in order to convert
   web URIs to model URIs in the database or local file names.

   E.g. if you have a model in the RAP database with the following URI:

   ```
   http://example.com/2004/testmodel1
   ```

   and you want to access it via the netapi endpoint at the following web URI:

## Using the NetAPI

### Querying

**1. Downloading whole models**

The easiest way to get started is to point an html browser at the URI endpoint
for the model. E.g. for the above example, use

`GET http://myhostedwebspace.net/rdfapi-php/netapi/testmodel2
HTTP/1.1`

  

**2. Fetch Queries**

The fetch query language returns all known information about a URIref. A query
consists of single "r" parameter, whose value is the URIref. The exact extent
of the RDF returned is dependent on the server. The NetAPI searches for all
statements with the resource as subject, then calculates the bNode closure of
all objects of these statements.

| Parameter Name | Parameter Value | Description |
| --- | --- | --- |
| lang  (required) | Id. of query language | URI: http://jena.hpl.hp.com/2003/07/query/fetch  Short forms: "fetch" |
| r  (required) | URIref | URIref of the resource |

  
Example fetch query:  
  

`GET http://myhostedwebspace.net/rdfapi-php/netapi/testmodel2?lang=fetch&r=http://example.com/employees/BillParker/
HTTP/1.1`

**3. RDQL Queries**

See also: The RAP RDQL Tutorial

| Parameter Name | Parameter Type/Value | Description |
| --- | --- | --- |
| lang  (required) | Id. of query language | URI: http://jena.hpl.hp.com/2003/07/query/RDQL  Short forms: "RDQL", "rdql" |
| query  (required) | The query, as %encoded string | The RDQL query to be executed.  As with all parameter values, this must be %-encoded. |
| closure  (optional) | "true" or "false" | For each binding of a variable, which is bound to a bNode, the graph is augmented with the bNode closure from this point. |

Example RDQL query:

`GET http://myhostedwebspace.net/rdfapi-php/netapi/testmodel2?lang=rdql&  
query=SELECT%20*%20WHERE%20(?x,<http://www.w3.org/2001/vcard-rdf/3.0/EMAIL>,?y)(?y,?z,"M.Murphy@example.com")  
&closure=true HTTP/1.1`

**4. SPO Queries**

SPO (also known as "Triples" or "find(SPO)") is an experimental minimal
query language. An SPO query is a single triple pattern, with optional subject
(parameter "s"), predicate (parameter "p"), and object (parameter "o", if a
URIref, parameter "v" for a string literal). Absence of a parameter implies
"any" for matching that slot of the triple pattern.

| Parameter Name | Parameter Value | Description |
| --- | --- | --- |
| lang  (required) | Id. of query language | URI: http://jena.hpl.hp.com/2003/07/query/SPO  Short forms: "SPO", "spo" |
| s  (optional) | URIref | URIref of the subject of the triple pattern.  Absence implies a wildcard match. |
| p  (optional) | URIref | URIref of the predicate of the triple pattern.  Absence implies a wildcard match. |
| o  (optional) | URIref | URIref of the object of the triple pattern.  Only one of "o" and "v" may be given |
| v  (optional) | String | Literal value as a string.  Absence or "o" and"v" implies a wildcard match. |
| closure  (optional) | "true" or "false" | Calculate the bNode closure of the matched subgraph. |

Example SPO query:

`GET http://myhostedwebspace.net/rdfapi-php/netapi/testmodel2?lang=spo` `& p=http://www.w3.org/2001/vcard-rdf/3.0/Family  
& v=Parker` `& closure=true HTTP/1.1`

### Serialization Formats

The netapi supports content type negotiation to determine the output RDF serialization
format. E.g. to get the results of a fetch query in n3 format, do:

```
GET /rdfapi-php/netapi/testmodel?lang=fetch&r=http://example.com/myterm HTTP/1.0
Accept: application/n3
```

The current implementation supports the following content mime types:

- application/rdf+xml
- application/n3
- application/n-triples
- text/xml

### Adding and Removing Subgraphs

To add and remove subgraphs from the store you need to send POST
requests with an 'op' argument of 'add' or 'remove' and the rdf to
add/remove in the body of the request. E.g.:

```
POST /rdfapi-php/netapi/testmodel?op=remove
Content-type: application/n-triples
Content-length: 353
...RDF...
```

N.B. most http apis will add the content-length header for you, but you'll
need to set the Content-type to be one of the above mime types.

Add and remove can be enabled in the config.inc by setting NETAPI\_ALLOW\_ADD
and NETAPI\_ALLOW\_REMOVE to true. They are disabled by delault.
